# Supplementary material for: The Evolution and Characterization of the RNA Interference Pathways in Lophotrochozoa
Source: Genome Biol Evol. 2024 May 7;16(5):evae098. doi: 10.1093/gbe/evae098 (PMC11114477; doi:10.1093/gbe/evae098)
Supplement: evae098_Supplementary_Data [file evae098_supplementary_data.zip › Supplementary_materials.zip!/selected_miRNAs/NoGe_selected_miRNAs/known_miRNA_families_pdf_files/NMRB01000087.1_Notospermus_geniculatus_strain_Ushimado_scaffold87,_whole_genome_shotgun_sequence_3152.pdf]

Provisional ID	:	NMRB01000087.1_Notospermus_geniculatus_strain_Ushimado_scaffold87,_whole_genome_shotgun_sequence_3152
Score total	:	2852.2
Score for star read(s)	:	3.9
Score for read counts	:	2843.4
Score for mfe	:	1.8
Score for randfold	:	0
Score for cons. seed	:	3
Total read count	:	5589
Mature read count	:	5583
Loop read count	:	0
Star read count	:	6

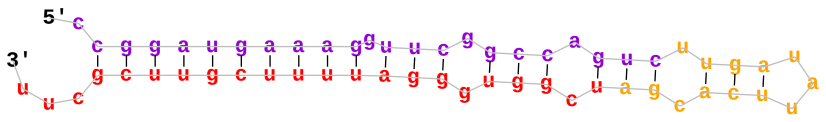

Genome coordinates: NMRB01000087.1 26378-26436

pre-miRNA sequence:
CCGGAUGAAAGGUUCGGCCAGUCCCGGAUGAAAGGUUCGGCCAGUCUUGAUAUUCACGAUCGGUGGGAUUUUCGUUCGCUU

Mature sequence: UCGGUGGGAUUUUCGUUCGCUU

Aligned mature sequence of the *N. geniculatus* pre-miRNA with the top 5 hits from the BLAST search against the pre-miRNA mirGeneDB set:

```
                              ******************* *
Nge_Mir-278                   ucggugggauuuucguucgcuu
Efe-Mir-278-P2_3p             ucggugggauuuucguucguuc
Lan-Mir-278_3p                ucggugggauuuucguucgguc
```
